# Supplementary material for: Polaramycin B, and not physical interaction, is the signal that rewires fungal metabolism in the Streptomyces–Aspergillus interaction
Source: Environ Microbiol. 2022 Jul 18;24(10):4899–914. doi: 10.1111/1462-2920.16118 (PMC9796313; doi:10.1111/1462-2920.16118)
Supplement: Supplementary file 1 — FIGURE S1 Top/Right: Experimental setup with an example of the confrontation assay on agar plates and a magnified section of it at the left side of the plate photograph. The raster with numbers represent areas that were cut out from the agar and analyzed by mass spectrometry (see Experimental procedures). Growing S. rapamycinicus cells are indicated by blue line circled areas, the A. nidulans colony is indicated by green line circled areas; dotted green line shows the extent of fungal growth (complete right hand side of plate is covered by fine mycelia). Dotted black circles indicate the areas of AMM or M79 media in the plate. Conidiation is strongest in the region facing the S. rapamycinicus colony. Heatmaps show metabolite concentrations found in the respective regions, positions of colonies are indicated by blue and green lines. Abbreviations: Terra: terragine: Ors: orsellinic acid; Lecanoric: lecanoric acid; meth‐Ors: methylorsellinic acid; Viol I: violaceol I; Viol II: violaceol II; Nig: nigericin; EmA, EmC, EmE: emericellamid ‐A, ‐C, ‐E; Stc: sterigmatocystin, dh‐Stc: dihydrosterigmatocystin, seco‐Stc: secosterigmaticystin; Aust: austinol; dh‐Aust: dihydro‐austinol; c‐Pro‐Tyr: cyclo(l‐Pro‐l‐Tyr); c‐Pro‐Val: cyclo(l‐Pro‐l‐Val) FIGURE S2 Spearman correlation plot of metabolites identified in the agar plate. Blue: positive correlation, red: negative correlation FIGURE S3 Agar plate test of S. zaomyceticus, S. ederensis, S. lateritius and S. europaeiscabiei for inducing yellow pigment production. Neither of these strains contains a homologous azalomycin F3a gene cluster FIGURE S4 Dose response of Polaramycin B treatment in respect to YP production (measured by absorption at 400 nm) in A. nidulans [file EMI-24-4899-s002.docx]

**Supplementary Figure S1: Experimental Setup and heatmap of metabolite levels**


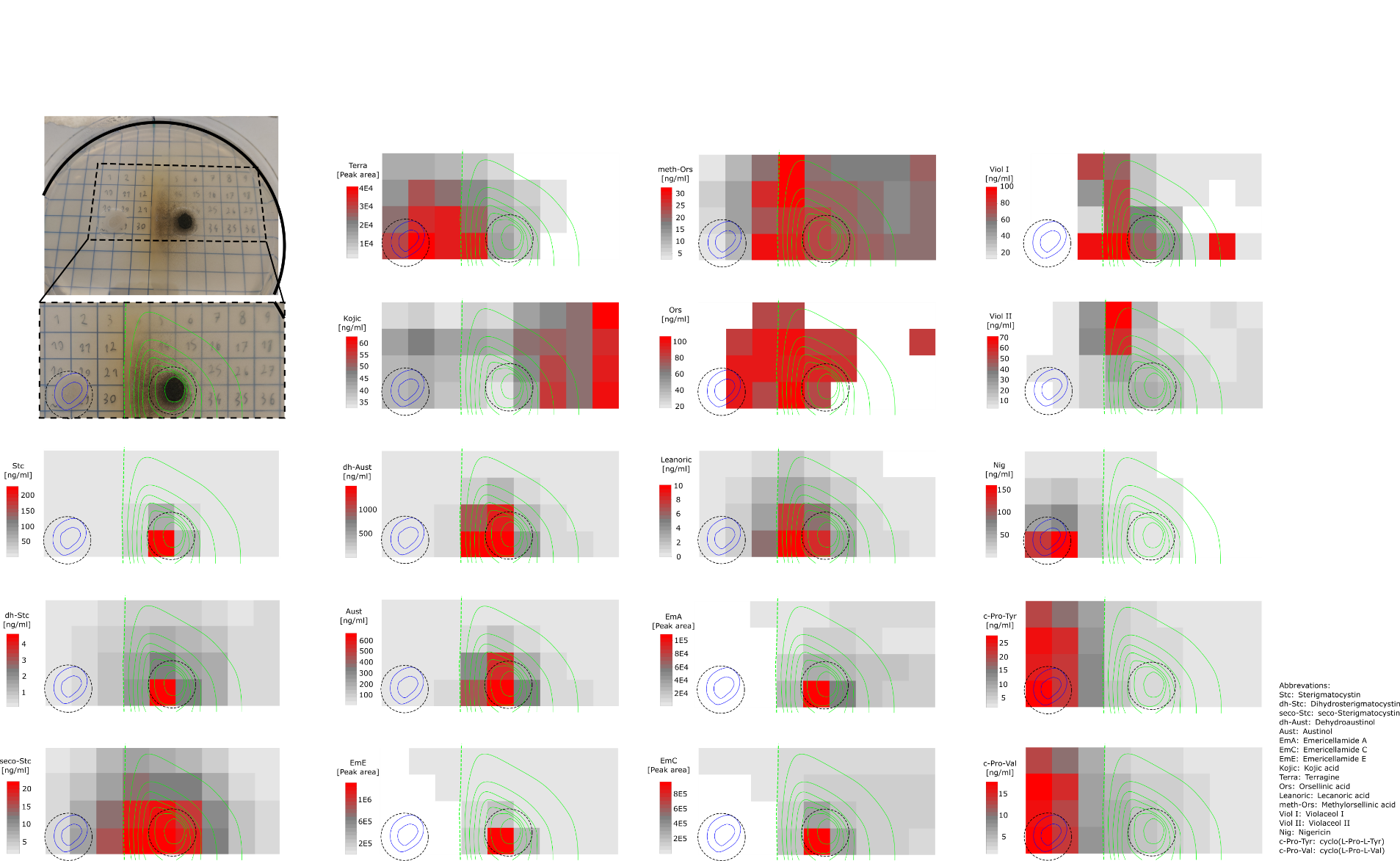


**Figure S1:** Top/Right: Experimental setup with an example of the confrontation assay on agar plates and a magnified section of it at the left side of the plate photograph. The raster with numbers represent areas that were cut out from the agar and analyzed by mass spectrometry (see materials and methods). Growing *S. rapamycinicus* cells are indicated by blue line circled areas, the *A. nidulans* colony is indicated by green line circled areas; dotted green line shows the extent of fungal growth (complete right hand side of plate is covered by fine mycelia. Dotted black circles indicate the areas of AMM or M79 media in the plate. Conidiation is strongest in the region facing the *S. rapamycinicus* colony. Heatmaps show metabolite concentrations found in the respective regions, positions of colonies are indicated by blue and green lines. Abbreviations: Terra: terragine: Ors: orsellinic acid; Lecanoric: lecanoric acid; meth-Ors: methylorsellinic acid; Viol I: violaceol I; Viol II: violaceol II; Nig: nigericin; EmA, EmC, EmE: emericellamid -A, -C, -E; Stc: sterigmatocystin, dh-Stc: dihydrosterigmatocystin, seco-Stc: secosterigmaticystin,; Aust: austinol dh-Aust: dihydro-austinol; c-Pro-Tyr: cyclo(L-Pro-L-Tyr); c-Pro-Val: cyclo(L-Pro-L-Val)

**Supplementary Figure S2**


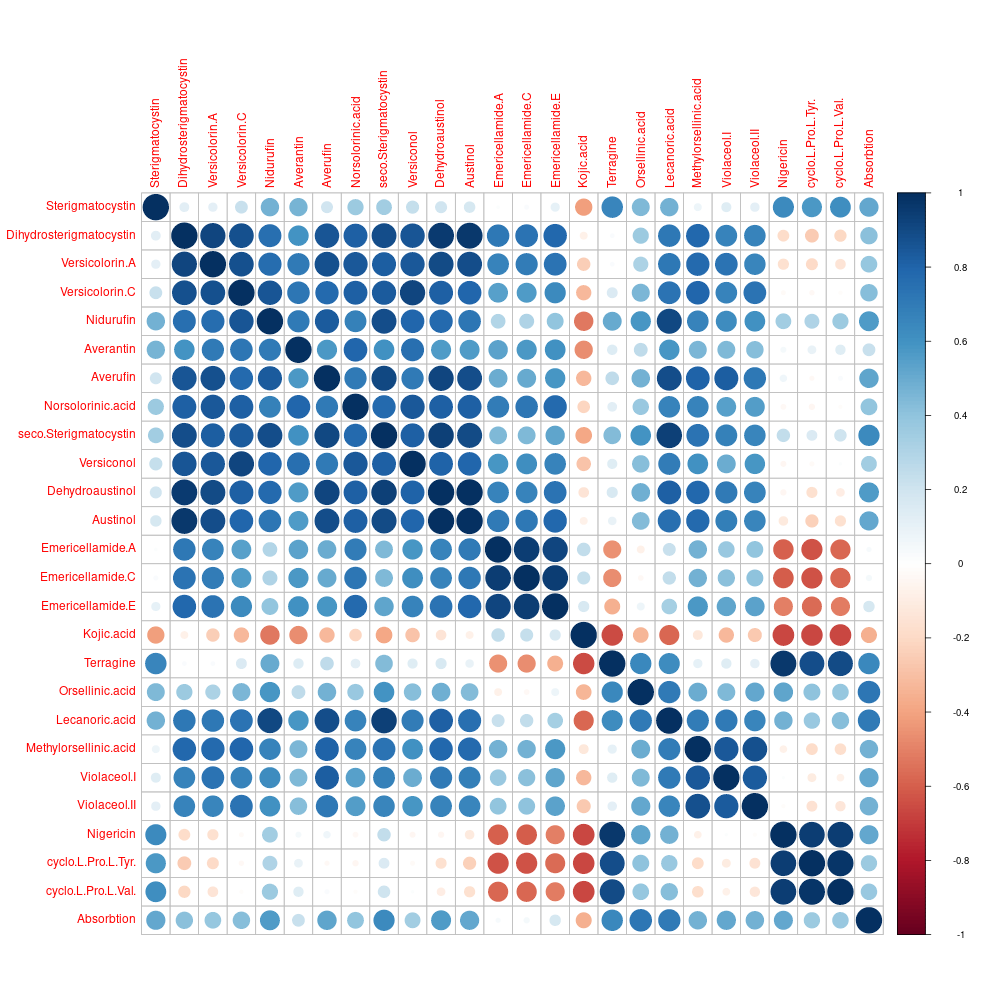


Figure S2: Spearman correlation plot of metabolites identified in the agar plate. Blue: positive correlation, red: negative correlation.

**Supplementary Figure S3**


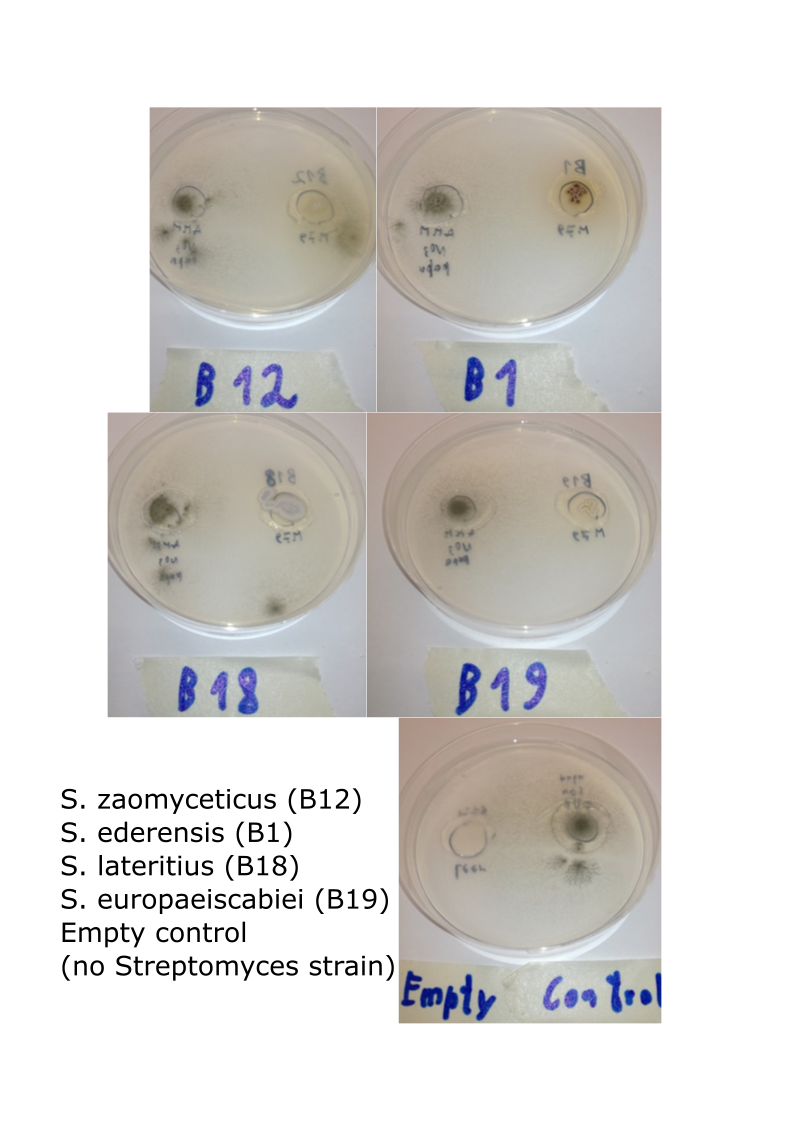


Figure S3: Agar plate test of *S. zaomyceticus*, *S. ederensis*, *S. lateritius* and *S. europaeiscabiei* for inducing yellow pigment production. Neither of these strains contains a homologous azalomycin F3a gene cluster.

**Supplementary Figure S4**


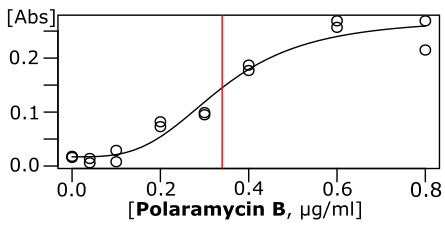


Figure S4: Dose response of Polaramycin B treatment in respect to YP production (measured by absorption at 400 nm) in *A. nidulans.*
